# When the skin speaks what HIV dictates: a series of particular cases of cutaneous manifestations in HIV

**DOI:** 10.1186/1471-2334-14-S7-P78

**Published:** 2014-10-15

**Authors:** Anca Răducan, Adina Alexandru, Sorin Rugină

**Affiliations:** 1 Colentina Clinical Hospital, Bucharest, Romania; 2 Carol Davila University of Medicine and Pharmacy, Bucharest, Romania; 3 Clinical Infectious Diseases Hospital of Constanța, Romania; 4 Faculty of Medicine, “Ovidius” University, Constanța, Romania

## Background

Recent data show that almost 75% of HIV patients have muco-cutaneous diseases, the proportion of patients with dermatoses being inversely proportional to the CD4 + and directly proportional to the stage of disease. Papulo-pustular eruption is the most common pruritic dermatosis in patients with HIV infection, followed by seborrheic dermatitis, psoriasis, molluscum contagiosum and drug reactions.

## Case report

We report a series of cases of patients with skin diseases associated with HIV infection, which are distinguished by particular features and clinical course.

Case 1: 54 years old patient with generalized psoriasis and HIV-syphilis co-infection. Case 2: male patient, aged 32 years, with acrodermatitis continua of Hallopeau and HIV infection. Case 3: 34 years old female patient with papulo-pustular acne, molluscum contagiosum, plantar warts, cutaneous xerosis and HIV-HBV co-infection. Case 4: female patient aged 26 years with 20 years history of HIV infection and epidermodysplasia verruciformis for 10 years. Case 5: 25 years old male patient with excoriated and overinfected warts, hepatitis B and HIV infection. Case 6: Patient with HIV infection, seborrheic dermatitis and post herpes zoster scar. Case 7: Patient with HIV infection and pityriasis versicolor.

## Conclusion

Most HIV patients can present with a variety of skin diseases throughout the evolution of HIV infection, as a result of achieved immunodeficiency or treatment. Patients undergoing HAART have a different clinical progress of HIV infections and therefore of dermatological manifestations, provided that the immune system’s functionality is restored.

## Consent

Written informed consent was obtained from all patients for publication of this Case report and any accompanying images. A copy of the written consent is available for review by the Editor of this journal.

